# Development of an Age Friendly Curriculum for Longitudinal Medical Student Clerkships in Rural Community Settings

**DOI:** 10.1093/geroni/igaf122.4099

**Published:** 2025-12-31

**Authors:** Alpha Scheel, Mengru Wang, Katherine Bennett, Aimee Verrall, Elizabeth Phelan

**Affiliations:** University of Washington, Seattle, Washington, United States; University of Washington, Seattle, Washington, United States; University of Washington School of Medicine, Seattle, Washington, United States; University of Washington / School of Medicine, Seattle, Washington, United States; University of Washington, School of Medicine, Seattle, Washington, United States

## Abstract

Most medical schools in the U.S. have minimal geriatrics training requirements, and few provide rural healthcare training. The University of Washington School of Medicine has a unique multi-state program that offers medical students opportunities to train across the WWAMI (WA, WY, AK, MT, ID) region, which is predominantly rural and medically underserved. Students can opt for longitudinal training at a rural and/or underserved site through the Targeted Rural and Underserved Track (TRUST) or the WWAMI Rural Integrated Training Experience (WRITE) program. Many students who train in these programs ultimately pursue careers in primary care and return to practice in a rural/underserved area; however, they lack focused training in geriatrics. The goal of our curriculum was to address this training gap. In the pilot phase, a needs assessment was performed via focus groups with 18 medical students in the WRITE program. Results identified a need for 1) templates for evaluating geriatric syndromes, 2) printable resources for patients with limited access to the internet, and 3) audio resources for students. These findings informed the development of four modules on the 4Ms of Age-Friendly Care: Mentation, Mobility, Medications, and What Matters. A multi-modal educational strategy was then implemented with short videos, podcasts, one-page clinical guides, and relevant standardized test practice questions. The refined curriculum was rolled out to one cohort of 18 medical students in August 2025. Next steps include evaluation of self-reported competence in the 4Ms and professional interest in caring for older adults in rural settings pre and post course.

